# Cyclodextrins, Surfactants and Their Inclusion Complexes

**DOI:** 10.3390/molecules30193944

**Published:** 2025-10-01

**Authors:** Ana Pilipović, Vesna Tepavčević, Dileep Kumar, Mihalj Poša

**Affiliations:** 1 Department of Pharmacy, Faculty of Medicine, University of Novi Sad, Hajduk Veljkova 3, 21000 Novi Sad, Serbia; vesna.tepavcevic@mf.uns.ac.rs; 2 Laboratory for Chemical Computation and Modeling, Institute for Computational Science and Artificial Intelligence, Van Lang University, Ho Chi Minh City 70000, Vietnam; kumar.dileep@vlu.edu.vn; 3 Faculty of Applied Technology, School of Technology, Van Lang University, Ho Chi Minh City 70000, Vietnam

**Keywords:** critical micellar concentration, micelle, inclusion complex, stoichiometry, detailed balance

## Abstract

Herein, a brief overview of the cyclodextrin structure is provided, along with its most important derivatives. The difference between the water molecules in the outer hydration shell of cyclodextrin and those in its hydrophobic cavities is discussed. The structural characteristics of surfactants, along with their structural differences, are presented. An insight into the formation of surfactant micelles was given in aqueous solution. A thermodynamic model for the formation of the inclusion complex between surfactants and cyclodextrin in a solution is presented, explaining the hydrophobic effect, which drives the formation of the inclusion complex at lower and room temperatures. The influence of the size of the cyclodextrin cavity and the structure of surfactants on the stoichiometry of the inclusion complex, as well as on the affinity of the surfactant to the hydrophobic cavity of cyclodextrin, is discussed. The most important experimental methods used to study the cyclodextrin-surfactant inclusion complex are listed.

## 1. Introduction

The phenomenon of spontaneous complex formation between cyclodextrin macromolecules and surfactants remains a highly relevant and actively researched topic. This is not only because these systems serve as exceptional models for studying the dynamics of supramolecular assemblies—surfactants are considered “ideal guests” that allow for systematic investigation of cyclodextrin complexation due to their tunable hydrophilic and hydrophobic segments—but also because inclusion complexes of surfactants and cyclodextrins have been shown to spontaneously self-assemble into more complex, highly hierarchical structures with numerous innovative applications [1,2]. In addition, intensive research is ongoing in the search for surfactants with improved properties, particularly those derived from natural sources. These efforts are continuously leading to the discovery of novel surfactant structures that exhibit synergistic interactions, enhanced surface activity, and superior aggregation behavior—all of which open up broad possibilities for creatively combining cyclodextrins and their derivatives with surfactants.

In this paper, we aim to provide an overview of the latest findings and insights regarding the interactions between cyclodextrins and surfactants in a solution, as well as to offer a perspective on the future directions in the study of these supramolecular structures.

## 2. Structure, Properties, and Chemical Modifications of Cyclodextrins

Since their discovery more than 130 years ago, cyclodextrins have been extensively investigated—from their structural characterization to practical applications and large-scale industrial production. Cyclodextrins are chiral, non-reducing cyclic oligosaccharides composed of D-glucopyranose units linked by α-(1→4) glycosidic bonds. The naturally occurring cyclodextrins that are obtained by enzymatic degradation of starch are industrially produced by cyclodextrin glucanotransferase (CGTase): α-cyclodextrin (αCD), β-cyclodextrin (βCD), and γ-cyclodextrin (γCD)—consist of six, seven, and eight D-glucopyranose units, respectively [3,4]. While these are the major products of the enzymatic process, small amounts of large-ring CDs are also formed [5] that have been isolated from complex CD mixtures by chromatography [6,7,8,9,10,11]. The synthesis and purification of larger CDs (more than nine glucopyranose units) are prepared using the enzymatic process employing recyclable templates [12,13,14]. However, their inclusion complex-forming ability is limited [15,16]. Cyclic oligosaccharides with 9–12 glucose units have been designated as δ-, ε-, κ-, and μ-cyclodextrins, and the nomenclature can be extended [17]. The synthesis of cyclodextrins containing five glucopyranose units (CD5) has been reported [18], while chemical synthesis of CD4 and CD3 was accomplished by introducing specific bridging groups connecting the O-3 and O-6 positions of D-glucose [19,20].

**Figure 1:**
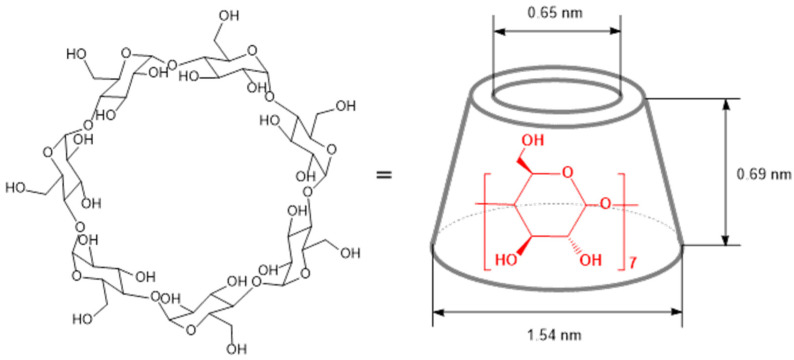
Structure and schematic model of β-cyclodextrin (βCD).

Cyclodextrins are biocompatible and biodegradable materials; in acidic solution, they undergo enzymatic hydrolysis by α-amylase to glucose, with the degradation rate following the order: γCD > βCD > αCD. Structurally, they are asymmetric toroidal molecules with a truncated cone shape, having a wider and a narrower rim. This shape results from the 4C_1_ chair conformation of the glucopyranose units [21]. The primary hydroxyl groups attached to the C6 carbon of each glucopyranose unit are located on one edge of the cyclodextrin ring (the narrower rim), whereas the secondary hydroxyl groups at the C2 and C3 positions are situated on the opposite, wider rim. The greater spatial freedom of the primary hydroxyl groups allows for free rotation, effectively decreasing the inner cavity diameter. All hydrophilic groups are concentrated around the openings of the cavity and are oriented outward, resulting in a highly hydrophilic exterior surface of the truncated cone structure. The interior of the cavity is lined with hydrogen atoms (C–H bonds) and glycosidic oxygen bridges, imparting a predominantly hydrophobic and apolar character to the internal environment [22]. Specifically, the non-bonding electron pairs of the glycosidic oxygen atoms are directed toward the cavity’s interior, increasing the electron density and conferring Lewis base character to the cyclodextrin cavity [1]. The number of glucopyranose units determines the diameter of the internal cavity: α-cyclodextrin has an approximate internal diameter of 0.50 nm, β-cyclodextrin around 0.65 nm, and γ-cyclodextrin about 0.80 nm [1] (Figure 1).

Natural cyclodextrins are soluble in water. Most of their chemical reactions are carried out in aqueous solutions and other suitable solvents for cyclodextrins are dimethyl sulfoxide, dimethylformamide, and pyridine, while they are generally insoluble in most organic solvents [23]. Among the natural cyclodextrins, βCD is the least water-soluble due to the formation of intramolecular hydrogen bonds between the hydroxyl groups of adjacent glucose units. Specifically, in βCD, the hydroxyl groups at the C2 and C3 positions of neighboring α-D-glucopyranosyl units are oriented in a way that maximizes their mutual interactions [24,25], making them less available for hydration compared to other cyclodextrins. Water solubility is more strongly influenced by the interaction of hydroxyl groups with water than by the internal hydrogen bonding between them. In contrast, the α-cyclodextrin ring is more strained, which results in greater spatial separation of its hydroxyl groups, rendering them more accessible for interaction with water molecules and contributing to its relatively higher solubility [24,25].

To improve their solubility and complexation capacity, various cyclodextrin derivatives are synthesized by functionalizing the hydroxyl groups located at the C2, C3, and C6 positions of the glucose units. These three hydroxyl groups exhibit different reactivities, allowing for selective chemical modifications [26,27]. The hydroxyl group at position C6 is the most basic and most accessible [28,29], the hydroxyl group at C2 is the most acidic, while the hydroxyl group at position C3 is sterically hindered. The list of the most common cyclodextrin modification reactions is presented in Table 1.

The most common cyclodextrin derivatives include hydroxypropylated CDs, carboxymethylated CDs, randomly methylated CDs, and sulfobutylether CDs [15]. Cyclodextrin substitution reactions involve the conversion of hydroxyl groups into other functional groups (deoxy-functionalized cyclodextrins) or reactions with hydroxyl groups leading to substitution at the oxygen atom of the hydroxyl group [30,31,32,33]. The most common and most accessible substitutions are mono-substitutions at the hydroxyl group at position 6, while secondary hydroxyl groups at positions C-2 and C-3 are more difficult to selectively modify [34]. Selective substitution at the C-2 hydroxyl group gives unsatisfactory yields and non-uniform products, whereas selective substitution at the hydroxyl group on the C-3 position is achieved by forming an inclusion complex of the reagent with cyclodextrin, orienting the reactive site toward the hydroxyl group at the C-3 position for reaction [35]. Mono-6-substituted cyclodextrins are synthesized via the formation of 6-O-aryl/alkyl-sulfonated CDs as intermediates, which are then further modified [30], most commonly into highly reactive 6-deoxy-6-halogenated CDs. The 6-O-aryl/alkyl-sulfonated CDs can also be processed into target products such as mono-azides, amines, or thiols [30,31,32,33], which can be used directly [36]. Di-6-deoxy-substituted CDs can also be synthesized with high selectivity into di-6-deoxy-6-thiolated α-CDs, which form intra- and intermolecular disulfide bridges [37]. Per-6-halogenated-per-6-deoxygenated CDs are synthesized in a first step and can be further modified in a second substitution reaction with thiourea to obtain per-6-thiolated CDs [38]. Randomly substituted CDs contain multiple functional groups at different positions (C-2, C-3, and/or C-6) [33,39]. The high degree of random thiolation can be achieved in the reaction of native CDs with thiourea followed by hydrolysis of the thiouronium ion by using a microwave reactor [38,40]. Per-modification of CDs results in the derivatization of all hydroxyl groups and this approach is most commonly used to improve solubility. Phosphorus pentasulfide was also used to prepare per-deoxy-thiolated β-CD in a one-step reaction [41]. The most important O-substituted CDs are methylated and hydroxypropylated derivatives, due to their high water solubility and complexation ability. Per-6-O-(tert-butyldimethylsilylated) CDs serve as protected intermediates for selective modification at the C-2 and C-3 positions, followed by a deprotection step [33]. This method is widely used for per-6-methylation of β-CD [42], and a simpler one-step method for the synthesis of per-6-methyl β-CD has also been developed using oligosaccharides complexed with CuSO_4_ [43]. Random substitution of hydroxyl groups at different positions is also possible and randomly methylated β-CD is one of the most important methylated derivatives.

CD hydroxyl groups can also participate in condensation reactions. The most important derivatives are 2-hydroxypropylated CDs. Among them, 2-hydroxypropyl-β-CD is the most studied due to its high water solubility, low toxicity, and strong complexation ability [44,45]. Hydroxypropylation of CDs is achieved via a condensation reaction between native CD and propylene oxide [46]. Polycondensation of CD with epichlorohydrin or polyepoxide compounds leads to branched polymeric materials with tunable solubility [47,48]. Random methylation of β-CD hydroxyl groups increases its water solubility up to 500 g/L, while hydroxypropylation at C-6 positions can raise it above 600 g/L [49]. Introducing ionic groups into the CD structure can further enhance water solubility. CDs can also be modified via esterification reactions with carboxylic acids. This reaction is commonly used for drug or targeting molecule conjugation to CDs, formation of CD-based surfactants [50], or enhancing the solubility of certain supramolecular CD-based structures [51,52]. Per-acetylated CDs, which are highly soluble in supercritical carbon dioxide or organic solvents, can form host–guest complexes in non-aqueous environments [53,54,55].

Esterification of CDs can be carried out enzymatically via transesterification [56,57,58], or chemically using carbodiimide chemistry or acid chloride forms of carboxylic acids [59,60]. Another approach is the use of CDs as initiators for the ring-opening of lactones (such as lactide), yielding fully biodegradable macromolecular CD esters [61,62,63]. Polycondensation can also be achieved by esterifying CDs with citric acid, poly (acrylic acid), or pyromellitic dianhydride [64]. Depending on the reaction conditions, either soluble branched polymers or insoluble gels or nanosponges may form having important applications [65,66,67,68].

Oxidation of CDs with NaIO_4_ leads to derivatives containing aldehyde functional groups [69], with increased water solubility [70], and display a broad spectrum of antimicrobial activity [71]. Aldehyde groups can further undergo reductive amination to produce cationic substructures with high water solubility [72,73]. Reductive amination can also be used to form bridged CD derivatives [74]. Polymeric derivatives of β-CD have also been developed, such as polyethylene glycol-βCD and dextran-βCD [2]. Cyclodextrin-based polyrotaxanes consist of polymer chains threaded through cyclodextrin rings [75]. To prevent the CDs from slipping off the polymer axis, stopper molecules are added to the chain ends that can be sensitive to external stimuli, allowing controlled degradation of the polyrotaxane under specific conditions. As a result, this class of materials represents a promising tool for various biomedical applications [75,76,77].

Due to their toroidal architecture and distinct physicochemical features—including the coexistence of hydrophilic outer surfaces and a hydrophobic internal cavity, localized electron-rich regions, and the presence of highly ordered water molecules within the cavity—cyclodextrins are classified as cage-type host molecules. In aqueous media, they are capable of forming inclusion complexes with hydrophobic guest molecules or hydrophobic moieties via non-covalent interactions, thereby altering the guests’ physicochemical and biological profiles [78,79,80,81,82].

## 3. Surfactants: Properties, Behavior, and Structural Diversity

**Figure 2:**
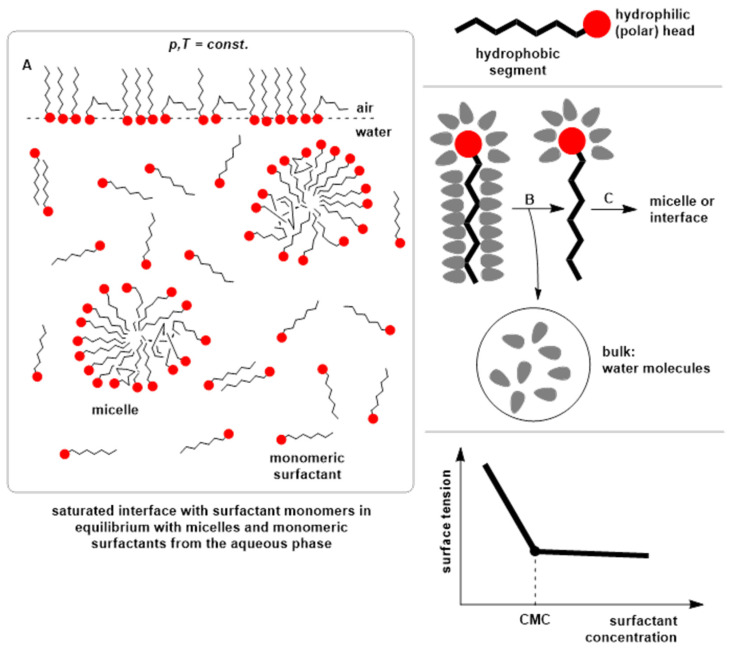
Schematic representation of surfactant self-assembly and micellization. (A) At constant temperature and pressure, surfactant monomers distribute between the bulk solution, the air–water interface, and micelles once the concentration exceeds the critical micellar concentration (CMC). The hydrophilic (polar) head groups (red circles) are oriented toward the aqueous phase, while hydrophobic chains aggregate in the micelle core. (B) Surfactant molecules can locate at the micelle surface or the air–water interface, stabilized by hydrophobic and electrostatic interactions. (C) Water molecules structure around hydrophobic moieties, and their release upon micelle or inclusion complex formation contributes to the driving force of assembly. The lower panel illustrates the typical decrease in surface tension with increasing surfactant concentration, showing the breakpoint at the CMC.

Surfactants are substances that exhibit the ability to spontaneously accumulate at the interface between two phases (one phase is hydrophobic while the other is hydrophilic) and to self-assemble into colloidal aggregates, thereby reducing surface tension. For this reason, they are referred to as surface-active agents. When surfactant molecules are present in water as monomers, water molecules arrange themselves orderly around the hydrophobic portion of the surfactant, resulting in a decrease in the overall entropy of the system. Through spontaneous adsorption of surfactant molecules at interfaces or through self-association into various microstructures—most commonly micelles—the structured water molecules surrounding the hydrophobic part of the surfactant are released, leading to an increase in overall entropy (Figure 2)—usually at lower temperatures and around room temperature. Therefore, in an aqueous solution at relatively lower temperatures, the formation of micelles is driven by an entropic effect. In comparison, at relatively higher temperatures, micelles form due to an enthalpic-driven force. Namely, with the increase in temperature, the molar entropy of water in the hydration shell around the monomeric surfactant becomes equal to the molar entropy of water from inside the solution. At temperatures of 350–380 K, the entropy change during micellization is usually zero. Conversely, with increasing temperature, the van der Waals attractive interactions between hydrocarbon segments of surfactants in micellar particles increase, resulting in the release of energy (enthalpy) [83,84,85]. To exhibit these properties, in general, surfactants have structural elements that are lyophobic, i.e., weakly attracted by surrounding molecules (e.g., solvent molecules), and lyophilic parts that are strongly attracted by them. In practice, aqueous solutions of surfactants containing hydrophobic and hydrophilic segments are of the greatest significance [86].

**Figure 3:**
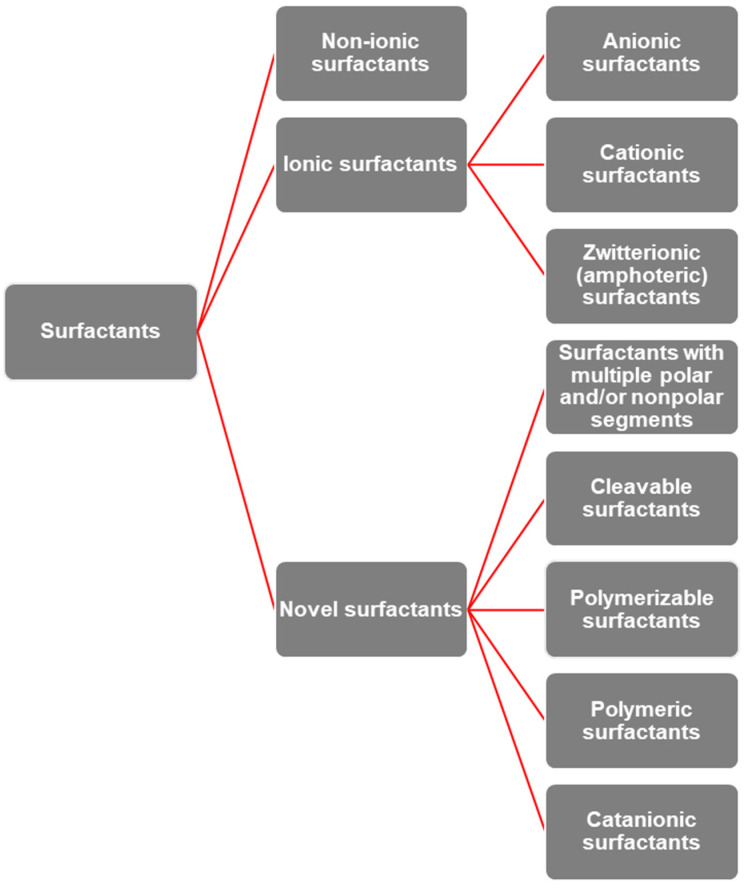
Classification of surfactants according to their dissociation in aqueous solution. Conventional surfactants are grouped into non-ionic and ionic types (anionic, cationic, and zwitterionic), while novel classes include cleavable, polymerizable, polymeric, and catanionic surfactants, as well as those with multiple polar/nonpolar segments.

There is no single, universally accepted classification of surfactants; instead, multiple categorizations exist [87]. One common approach is based on their applications: emulsifiers, foaming agents, wetting agents, dispersing agents, etc. However, this classification provides little insight into their chemical composition or other potential applications, especially since many surfactants exhibit multifunctionality. Another approach is based on physical properties such as solubility in polar or nonpolar solvents, stability under extreme conditions, and so on. A relevant classification is also based on their origin—whether of natural or synthetic (chemical) origin. The simplest and most scientifically accepted classification is based on the type of dissociation in water, i.e., on the nature of the hydrophilic portion or polar group [88]. This classification, presented in Figure 3, is considered the most useful, as it allows a straightforward correlation between chemical structure and surface activity, thereby enabling the development of general structure–property relationships for surfactants.

The hydrophobic part of a surfactant molecule typically consists of hydrocarbon segments, most commonly a long alkyl chain containing more than ten carbon atoms. The hydrophilic part may be represented by either polar non-ionic or ionic groups, based on which surfactants are classified as non-ionic or ionic.

Non-ionic surfactants exist in solution as electrically neutral molecules [89]. They represent the second largest class of surfactants (after anionic ones) and are generally compatible with all other types of surfactants. Ionic surfactants dissociate in aqueous solution into pairs of anions and cations, although typically only one of these ions exhibits surface activity. The ion of opposite charge is referred to as the counterion. Based on the charge and the nature of the ion responsible for surface activity, ionic surfactants can be further divided into [90,91]: anionic, cationic, and zwitterionic (or amphoteric) surfactants.

Most ionic surfactants are monovalent, although there are notable examples of divalent anionic amphiphiles. In the case of ionic surfactants, the choice of counterion influences their physicochemical properties. Most anionic surfactants have sodium as the counterion, while other cations—such as lithium, potassium, calcium, and protonated amines—are employed for specific applications. The counterions of cationic surfactants are typically halides or methyl sulfate [92].

Anionic surfactants dissociate in water into an amphiphilic anion, i.e., a negatively charged surfactant ion (such as carboxylate, sulfate, sulfonate, carboxybetaine, sulfobetaine, or quaternary ammonium), and a cation, i.e., a positively charged ion, most commonly a small alkali metal ion (sodium, potassium, calcium, magnesium) or a quaternary ammonium ion [93]. Anionic surfactants represent the largest class of surfactants [90].

Cationic surfactants dissociate in water into an amphiphilic cation, i.e., a positively charged surfactant ion, and an anion, most commonly a halide. They represent the third largest class of surfactants and are generally incompatible with anions (with some exceptions).

Zwitterionic surfactants contain both anionic and cationic charges under normal conditions. In the literature, they are often referred to as amphoteric surfactants, which can behave as cationic, zwitterionic, or anionic species depending on the pH. Amphoteric surfactants contain two oppositely charged groups. Upon dissociation, the molecule yields both an amphiphilic anion and an amphiphilic cation [90].

Surfactants can also be distinguished by the nature and length of the hydrophobic part, which typically consists of a medium- or long-chain hydrocarbon (more than eight carbon atoms), which may be linear or branched, saturated or unsaturated.

Novel surfactant structures can be classified into the following groups [88,90,94,95]: surfactants with multiple polar and/or nonpolar segments, cleavable surfactants, polymerizable surfactants, polymeric surfactants, and catanionic surfactants. Surfactants with multiple polar and/or nonpolar segments represent more complex structures that may contain two hydrophobic tails and/or two polar head groups connected by a short linker (bridge) [75,79]. These surfactants include several different subclasses [88,90,94]: double-headed surfactants, double-tailed surfactants, gemini surfactants, bolaform surfactants, shamrock surfactants, and DOWFAX surfactants. Gemini surfactants can be considered surfactant dimers, i.e., two amphiphilic molecules connected by a so-called spacer.

**Figure 4:**
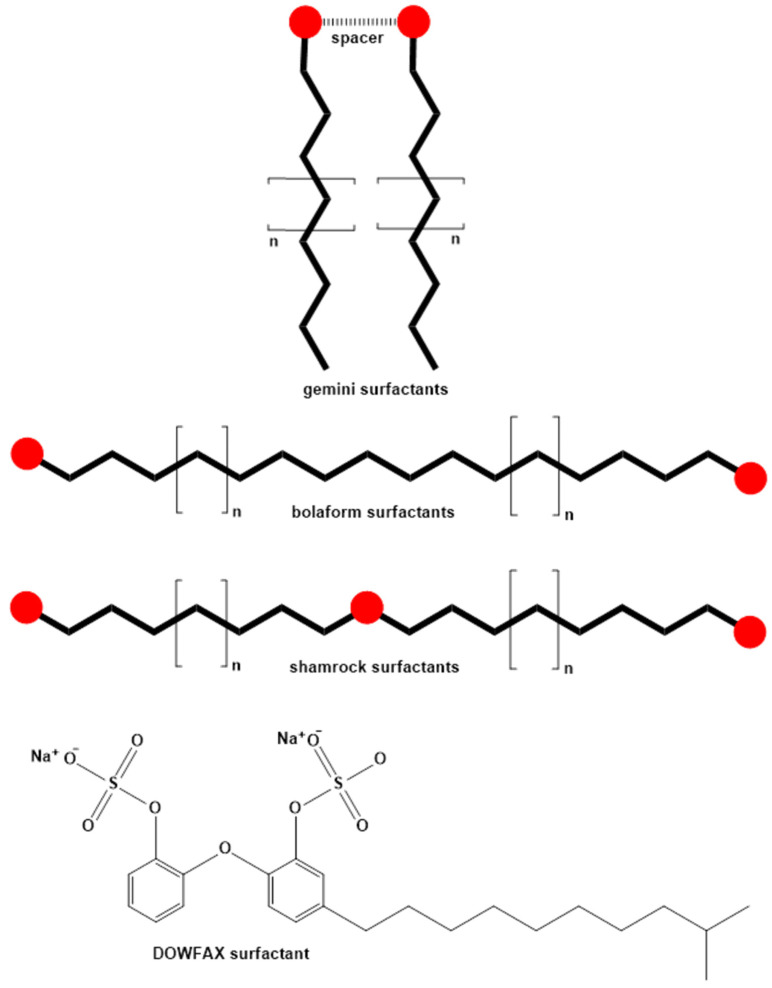
General structures of some surfactants and an example of DOWAX surfactant.

These surfactants have three distinct structural units: a hydrophilic group, a hydrophobic group, and a spacer group linking the two parts of the gemini surfactant, whose variability influences the surfactant’s properties [88]. Connecting the two surfactant halves at the ends of their hydrophobic tails results in the formation of so-called bolaform surfactants, whose physicochemical properties differ significantly from those of gemini surfactants [88,90,94,96]. Most gemini surfactants consist of two identical halves and are referred to as dimeric surfactants, but asymmetric gemini surfactants also exist, which either have tails of different lengths, different types of polar heads (heterogemini surfactants), or both [90]. Shamrock surfactants represent a novel class that contains a central head group connected to two flanking head groups by hydrocarbon chains; notably, they do not contain long-chain alkyl groups [97]. DOWFAX surfactant components are mono- and di-alkylated diphenyloxide disulfonates and mono- and di-sulfonated alkyl diphenyloxides [98] (Figure 4).

Cleavable surfactants represent a group of amphiphiles containing acid- or base-labile bonds: surfactants unstable in acidic media (cyclic acetals, alkyl glycosides, acetal oxo compounds, and ortho esters), surfactants unstable in basic media (quaternary ammonium compounds—quats, betaine esters, and other esters). Other types include surfactants with UV-labile bonds and ozone-degradable surfactants. Photo-labile surfactants contain diazosulfonate as the polar head group, surfactants with two hydrophobic chains, and Co(III) complexes susceptible to reduction. Ozone-degradable surfactants contain unsaturated bonds that readily degrade during water ozonization [90].

Polymerizable surfactants are an example of so-called functional surfactants, i.e., surfactants possessing additional properties beyond surface activity. Interest in polymerizable surfactants arises from the fact that surface activity may be necessary at one stage but unwanted or even detrimental in a subsequent stage of a process [88,90]. The main characteristic of these surfactants is the presence of a polymerizable reactive group, which can be located on either the polar hydrophilic or the hydrophobic part of the surfactant molecule [88]. These surfactants have the ability to undergo polymerization with other components in the system [90]. Polymeric surfactants are polymers exhibiting surface activity, also referred to as surface-active polymers. Their hydrophilic segment is typically a polysaccharide, with or without charge [88,90]. They can be: polymers with a hydrophilic backbone and hydrophobic side chains, polymers with a hydrophobic backbone and hydrophilic side chains (glycoproteins are natural representatives of this group, although their polypeptide backbone is not completely hydrophobic), or polymers with alternating hydrophilic and hydrophobic blocks.

Special surfactants are those capable of drastically reducing surface tension. Two types of special surfactants in this group are silicone surfactants and fluorinated surfactants. The original type of fluorinated surfactants contained polydimethylsiloxane as the nonpolar segment, whereas later representatives contain fluorocarbon and hydrocarbon groups as the hydrophobic part. Silicone surfactants are high-molecular-weight compounds and belong to polymers. Catanionic surfactants are equimolar mixtures of cationic and anionic surfactants (without inorganic counterions).

## 4. Thermodynamics of Inclusion Complex Formation Between Surfactants and Cyclodextrins

Inclusion complexes are supramolecular assemblies formed when cyclodextrin molecules, acting as hosts, encapsulate hydrophobic segments of guest molecules (such as surfactants) within their hydrophobic cavities. These complexes are driven by non-covalent interactions [99]. The driving forces governing the formation of cyclodextrin–guest inclusion complexes cannot be simply attributed to van der Waals (VDW) interactions [100]. Such forces are present both in bulk aqueous solution and within the CD cavity, and their contribution is often diminished when the guest does not ideally fit the hydrophobic interior of the CD [100]. A more accurate description emphasizes the role of hydrophobic effects, which occur in two distinct forms: classical [100,101] and non-classical [102,103]. The classical hydrophobic effect arises from the entropic gain associated with the release of high-energy water molecules from the CD cavity upon guest inclusion [101,102]. In contrast, the non-classical hydrophobic effect involves specific structuring of water molecules around the hydrophobic surfaces of the guest and the host, and has been shown to be highly relevant for many guest molecules, particularly surfactants [102,103]. These processes, in combination with possible hydrogen bonding and electrostatic contributions [100], provide a comprehensive framework for understanding CD–guest complexation. The investigation of interactions between cyclodextrins and guest molecules critically relies on their thermodynamic and stoichiometric characterization, which provides detailed insight into how and why these molecules associate. This characterization involves determining binding constants, stoichiometry, and thermodynamic parameters (enthalpy and entropy changes, as well as various derivatives of the Gibbs free energy of interaction), offering a concrete understanding that aids in formulation optimization, binding mechanism elucidation, and the design of innovative solutions across various industries.

There are two principal approaches for the determination of cyclodextrine surfactants inclusion complex formation thermodynamic parameters. In the first approach, the equilibrium constant is measured at different temperatures, and the enthalpy change (Δ*H*°) is subsequently derived from the van’t Hoff equation, with the entropy change (Δ*S*°) being calculated afterward. In contrast, isothermal titration calorimetry (ITC) provides, in a single experiment, the binding stoichiometry, the association constant, and the enthalpy change (Δ*H*°) [2].

**Figure 5:**
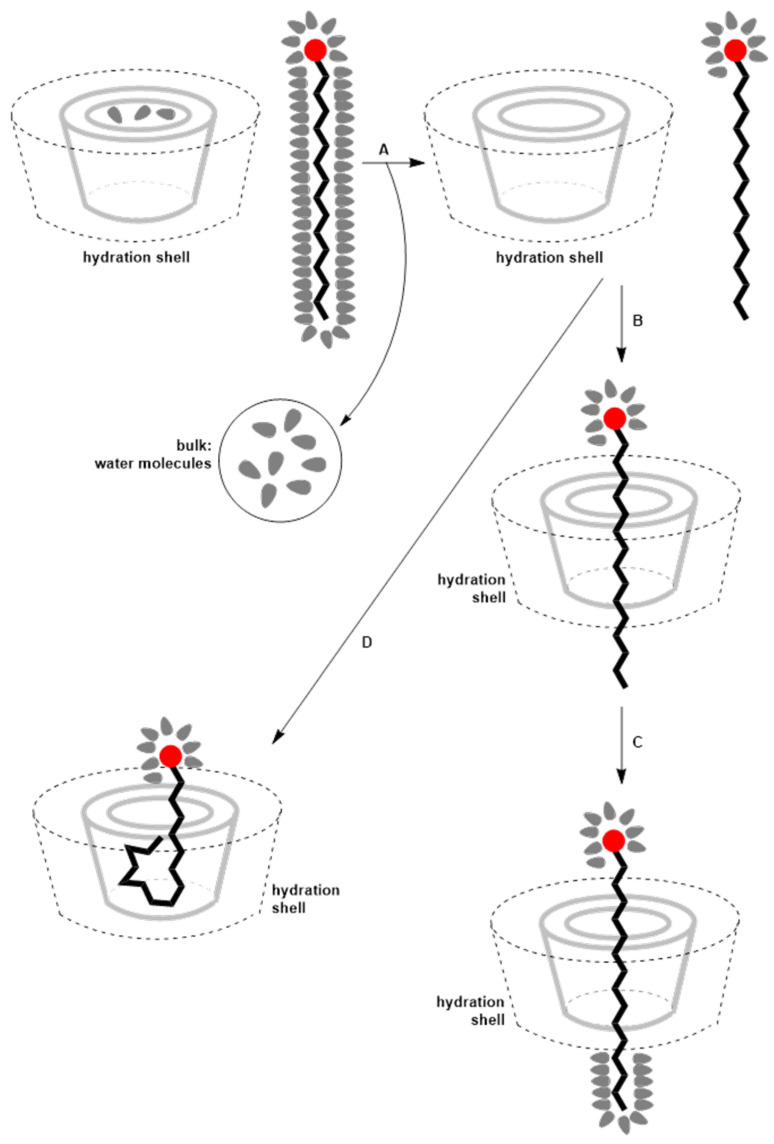
Formation of the cyclodextrin-surfactant inclusion complex: formal dehydration of the hydrophobic segment of the surfactant and the inner cavity of the cyclodextrin (A), the entry of the surfactant into the cavity of the cyclodextrin (B), the hydration of the hydrophobic segment of the surfactant that is not in the cavity of the cyclodextrin and the entry of a more or less twisted conformation of the hydrocarbon chain of the surfactant into the cavity of the cyclodextrin (D) when phase (C) does not occur.

The formation process of inclusion complexes between cyclodextrins and surfactants is complex and dynamic. It includes dehydration of both surfactants and cyclodextrins, inclusion of the surfactant into the cyclodextrin cavity, hydration of the inclusion complex, establishment of further equilibria, and supramolecular growth [2]. Unlike small molecule guests, surfactants possess extended hydrophobic tails and polar head groups, which introduce additional specific interactions such as steric accommodation within the CD cavity and headgroup–rim interactions, making their complexation behavior distinct from simple small molecules. Initially, high-energy water molecules are released. These water molecules are highly structured because they hydrate the surfactant’s hydrophobic part and the cyclodextrin’s hydrophobic and spatially confined internal cavity [104,105] (Figure 5). This high-energy water molecule release represents an entropy-favored process and is the main driving force for component association in aqueous environments [2]. For small molecules, the hydrophobic effect is simpler and often less directional, whereas surfactants require the cooperative alignment of their hydrophobic chains within the CD cavity, often accompanied by specific positioning of the polar head at the rim. The structure of water within the cyclodextrin cavity is influenced by the cavity size itself. Measurements of water heat capacity have shown that water in the cavities of β- and γ-cyclodextrins exhibits heat capacity values similar to bulk water, whereas in the smaller α-cyclodextrin cavity, water’s heat capacity decreases [106]. The amount of water hydrating the hydrophobic part of the surfactant depends on the size of the surfactant’s hydrophobic moiety. Subsequently, the hydrophobic part of the surfactant is allowed to enter the hydrophobic cyclodextrin cavity, stabilized primarily by van der Waals interactions [102,107]. During this process, interactions between the surfactant head group and the cyclodextrin rim are also present [108,109]. These headgroup–rim interactions are largely absent in complexes with small molecules, emphasizing the unique supramolecular architecture of CD–surfactant complexes. Thanks to these interactions, modulation of cyclodextrin substituent groups or use of cyclodextrin derivatives can influence the stabilization of inclusion complexes with surfactants [110,111]. Once the inclusion complex is formed, water molecules rehydrate the exposed parts of the guest molecule, integrating into the hydration shell of the complex.

Water plays a crucial role in the formation of inclusion complexes between cyclodextrins and surfactants. It has been demonstrated that the addition of co-solutes such as alcohols or salts to a cyclodextrin–surfactant system alters the behavior of water, thereby shifting the complexation equilibrium. The addition of short-chain alcohols (e.g., ethanol, propanol, butanol) leads to a decrease in the affinity between cyclodextrins and surfactants [112,113,114,115,116], as these alcohols act as hydrotropes and stabilize the surfactant alkyl chains. These surfactant chains would otherwise tend to insert into the cyclodextrin cavity to escape from the aqueous environment. Since the alkyl chains become more stabilized in solution, their driving force to enter the cyclodextrin cavity is reduced, resulting in a lower binding affinity and a decreased binding constant.

On the other hand, the addition of an inert salt (e.g., NaCl) does not involve direct interaction with either the cyclodextrin or the surfactant but modifies the solvent properties [117]. It increases the polarity of water, rendering it more “hostile” to the hydrophobic moieties (alkyl chains), which are thus driven to seek shelter within the hydrophobic cavity of the cyclodextrin. This enhances the hydrophobic effect and leads to stronger binding. Furthermore, the osmotic pressure increases, thereby pushing the equilibrium toward the formation of a less hydrated and more stable inclusion complex.

In aqueous solutions containing both cyclodextrin and surfactant molecules, micellization of the surfactant occurs simultaneously with inclusion complex formation. Throughout this process, there exists an equilibrium between free surfactant molecules and surfactant incorporated in micelles, which is influenced by the presence of cyclodextrins. The formation of inclusion complexes reduces the concentration of free monomeric surfactant available for micelle formation. However, the cyclodextrin-surfactant inclusion complex is not surface-active. In that case, i.e., it is not adsorbed on the water solution/air boundary surface, then based on the detailed balance theory, the equilibrium constant of micelle formation in the system without cyclodextrin is identical to the same constant if cyclodextrin is present in the surfactant aqueous solution [117]. Therefore, it follows from the thermodynamic analysis that neither the concentration of monomeric surfactant, i.e., the critical micellar concentration (CMC = *const*.) does not change in the presence of cyclodextrin. There is the same concentration of free monomeric surfactant when forming micelles in an aqueous solution with and without cyclodextrin. Of course, in the presence of cyclodextrins, the experimentally determined critical micellar concentration (CMC_exp_) is higher than CMC = *const*. values for the concentration of the monomeric surfactant (C_monomeric_) that is incorporated into the cyclodextrins cavity CMC_exp_ = CMC + C_monomeric_ (for the case of formation of an inclusion complex of stoichiometry 1:1). While the equilibrium concentration of cyclodextrin-surfactant inclusion complex of 1:1 stoichiometry (C_CD-S(eq)_) in aqueous solution is: CMC_exp_ − CMC = C_monomeric_ = C_CD-S(eq),_ and the equilibrium concentration of free cyclodextrin is: C_CD(T)_ − C_CD-S(eq)_ (C_CD(T)_ represents the total concentration of cyclodextrin). Therefore, the equilibrium constant of the formation of the inclusion complex of 1:1 stoichiometry is as follows [118,119]:

K = [CMC_exp_ − CMC]/CMC(C_CD(T)_ − [CMC_exp_ − CMC]) (1)

Moreover, inclusion complexes of cyclodextrins and surfactants can spontaneously organize into supramolecular structures via hydrogen bonding between hydroxyl groups on their external surfaces.

## 5. Cyclodextrins Host–Guest Complexes with Surfactants: Binding Constants and the Stoichiometry

Cyclodextrins form host–guest complexes with surfactants, exhibiting high binding constants, and the stoichiometry of these complexes depends on the alkyl chain length of the surfactant and the size of the CD cavity [112,113]. Most commonly, 1:1 complexes are formed, denoted as surfactant-CD, which typically occurs with surfactants having short alkyl chains (fewer than 8 carbon atoms). For surfactants with longer chains (12 or more carbon atoms), 2:1 complexes (surfactant-2CD) are usually formed. In the case of γ-cyclodextrin, due to its larger internal cavity, 1:2 complexes (2surfactant-CD) can form, especially when the surfactant is present in excess relative to the cyclodextrin [114,115,116]. Additionally, 4:1 complexes are specific to gemini surfactants [120].

For homologous surfactants, the binding constant increases significantly with the elongation of the hydrophobic tail [121,122,123,124], and this increase becomes much less pronounced for hydrocarbon chains longer than 14 carbon atoms, likely because the cyclodextrin cavity becomes “saturated” with a C14 chain.

For hydrocarbon surfactants, the binding constant with α-CD is significantly higher than with β-CD, as the hydrocarbon chain fits better into the smaller cavity of α-CD. Fluorocarbon surfactants bind very weakly to α-CD, but strongly to β-CD, since the fluorocarbon chain is too large for the α-CD cavity but fits well into the β-CD cavity. Xing and colleagues directly confirmed the preference of α-CD for hydrocarbon surfactants, and β-CD for fluorocarbon surfactants [125].

For ionic surfactants, the type of headgroup does not significantly affect the binding constants. However, for nonionic surfactants, a decrease in the binding constant has been observed with increasing length of the ethoxylate chains, which is presumed to be related to hydrogen bonding between the ethoxylate groups and water [126].

The binding constants of some gemini surfactants are significantly lower compared to their single-chain analogs [127,128], which has been explained by hydrophobic interactions and steric constraints between the two hydrocarbon tails within one gemini surfactant. Additionally, it has been shown that the binding constant increases with the lengthening of the spacer between the chains, as a result of the separation of the two tails [94].

The polar head has little influence on the stability of 1:1 stoichiometry complexes, although a slight decrease in stability has been observed when the SO_4_^2−^ group is replaced with cationic groups in surfactants with the same alkyl chain length. The effect of the surfactant head becomes more significant in 2:1 complexes, as the presence of two cyclodextrin molecules brings the surfactant headgroup closer to the CD opening. In such cases, if energetically favorable, additional interactions may occur that further stabilize and facilitate the formation of the inclusion complex [129].

Complexes with nonionic surfactants: in the case of ethoxylated alcohols, both the hydrophilic and hydrophobic parts can enter the α-CD cavity. Alkylphenol units do not form complexes with α-CD [130,131], but only with β-CD, which has a higher affinity for the bulky hydrophobic moiety [131,132]. As with ionic surfactants, it has been shown that γ-cyclodextrin (γ-CD) can also form a complex with two surfactant molecules within its large cavity [124]. The surfactants critical chain length for complex formation is 8 carbon atoms for nonionic surfactants and 6 carbon atoms for ionic surfactants [133,134,135,136].

The effect of counterions on the binding constants of complexes between ionic surfactants and cyclodextrins is small compared to the energetic changes caused by other processes [1]. However, potentiometric studies have shown small binding constants for counterions (e.g., bromide with C12TAB-βCD (inclusion complex of dodecyl-trimethyl ammonium bromide with β-cyclodextrin) and C12TAB-HPβCD (inclusion complex of dodecyl-trimethyl ammonium bromide with hydroxypropyl-β-cyclodextrin), and sodium with βCD) [137,138,139].

### 5.1. The Influence of Surfactant Concentration, Cyclodextrin and Temperature on the Formation of the Inclusion Complex and the Self-Association of the Inclusion Complex

It has been shown that inclusion complexes, depending on concentration and temperature, self-assemble into planar, bilayer aggregates—building blocks of lattice-like structures [113,114,115,116,120,140,141,142,143,144]. A typical arrangement involves one surfactant and two CDs, oriented “head-to-head” [116], and the assembly is governed by strong water-mediated hydrogen bonds, in contrast to the weak hydrophobic interactions present in conventional micelles [141,145,146]. Chemical modifications of cyclodextrins (such as hydroxypropylation and methylation) inhibit self-assembly, confirming the crucial role of hydrogen bonding in the formation of inclusion complex aggregates [141,143,147,148]. In studying the interactions between weakly acidic anionic alkyl ether carboxylates (which possess both ionic and nonionic surfactant characteristics) and α- and β-cyclodextrins, it was possible to systematically vary the charge of the surfactant (via pH adjustment) without altering its chemical structure. It was shown that these surfactants spontaneously form inclusion complexes with cyclodextrins, which can further self-assemble into supramolecular aggregates [149]. Electrostatic repulsion was demonstrated to play a key role in organizing these inclusion complexes—specifically, the presence of charge was found to be essential for the spontaneous formation of well-ordered, multilayered structures with a periodicity of approximately 10 nm. This structural ordering can be further tuned by selecting the appropriate cyclodextrin cavity size.

So far, it has already been discussed how the formation of the cyclodextrin-surfactant inclusion complex affects the value of the critical micellar concentration. Now the influence of the surfactant concentration above the experimentally determined critical micellar concentration (CMC_exp_) is observed. Surfactant at concentrations above the CMC_exp_, two simultaneous processes occur: complexation of surfactant monomers by CDs and the self-assembly of these monomers into micellar aggregates. The competition between these two processes leads to a significant concentration of free CD in equilibrium with the micellar aggregates, and the percentage of uncomplexed β-CD in equilibrium with the micellar system increases with the hydrophobicity of the surfactant molecules. In such systems, the auto-aggregation of surfactant monomers is more dominant than the complexation process, and by varying the hydrophobicity of the surfactant monomers, it has been found that the percentage of uncomplexed CD in equilibrium with the micellar system ranges from 5% to 95%. When the self-assembled structure of the surfactant is vesicular, the amount of free CD in the mixed CD–surfactant system can reach 100% [150].

By studying the effect of temperature on cyclodextrin–surfactant supramolecular aggregates, it was shown that these aggregates can be controllably transformed, which is important for the development of drug delivery carriers or other controlled release systems. The behavior of a system containing epoxy-β-cyclodextrin (EP-β-CD) and a mixture of anionic and cationic surfactants—sodium dodecyl sulfate (SDS) and dodecyltrimethylammonium bromide (DTAB)—in aqueous solution was investigated [151]. Depending on the temperature, the SDS/DTAB—EP-β-CD complex self-assembles into worm-like micelles at lower temperatures and into vesicles at higher temperatures. The key driving forces for this organization are hydrogen bonds between EP-β-CD molecules, which promote aggregate formation, and water molecules, which facilitate vesicle formation. To provide quantitative support to these general trends, detailed data from representative systems are summarized in Table 2 and briefly discussed below.

The quantitative data summarized in Table 2 provide a clearer picture of the key aspects of CD–surfactant complexation. In general, α-CD binds short- to medium-chain hydrocarbon surfactants more strongly than β-CD, with binding constants (logK) increasing with the alkyl chain length (e.g., C8: logK α-CD ≈ 2.6 vs. β-CD ≈ 1.9) [113,116]. For anionic surfactants such as SDS, the presence of β-CD significantly affects the aggregation process, shifting the critical micellar concentration from 8.2 mM to ~15 mM under equimolar conditions [140,144]. Cationic surfactants form relatively stronger complexes, with β-CD–DTAB showing logK ≈ 2.3 and β-CD–CTAB ≈ 3.1 [141,145].

The influence of surfactant concentration above the experimentally determined CMC (CMCexp) also reveals a strong competitive interplay between complexation and micellization. The percentage of uncomplexed β-CD in equilibrium increases with surfactant hydrophobicity, ranging from ~5% for C12 to ~95% for C16, and in vesicular systems may reach 100% [150]. For weakly acidic anionic alkyl ether carboxylates, varying the charge by pH adjustment leads to the spontaneous formation of supramolecular aggregates with multilayered periodicity (~10 nm), demonstrating the importance of electrostatic contributions [149]. Furthermore, temperature-dependent studies of EP-β-CD with mixed SDS/DTAB surfactant systems indicate a reversible structural transition from worm-like micelles (25 °C) to vesicles (50 °C), highlighting the role of hydrogen bonding and water-mediated interactions in driving aggregation [151].

### 5.2. Methods Applied to the Study of Cyclodextrin Surfactant Systems

Host–guest interactions between cyclodextrins and surfactants are investigated using various experimental techniques that provide direct insight into the structural and physicochemical properties of these complexes: conductivity measurements [1,127,152,153,154,155,156,157], surface tension determination [154,155,156,157,158,159], isothermal titration calorimetry (ITC) [1,148,149,150], density and sound velocity measurements [1,148,149], use of selective electrodes for surfactants [156,157,158,159,160], kinetic methods [155], nuclear magnetic resonance (NMR) [156], small-angle neutron scattering (SANS) techniques [155], dynamic light scattering (DLS) [153], and spectrophotometric methods [1,2,158]. In addition, for a more comprehensive understanding of inclusion complexes and the detailed interactions between cyclodextrins and their guest molecules, computational methods such as quantum chemical calculations and molecular dynamics (MD) simulations are also employed [158,159].

### 5.3. Applications of Cyclodextrin Inclusion Complexes

Cyclodextrin–surfactant systems can protect, transport, and deliver drugs and other active substances in a targeted manner, making them suitable for use in drug delivery applications in the pharmaceutical industry. Cyclodextrin inclusion complexes with drugs offer numerous advantages: cyclodextrins improve the solubility, physicochemical stability, bioavailability, and overall stability of drugs, protect them from degradation, and reduce irritation. They are also used to enhance the odor and taste of pharmaceutical formulations and to prevent incompatibilities by physically separating mutually incompatible components [161]. Their potential application also extends to chemistry and biotechnology for the development of nanoreactors—microscopically controlled environments for chemical reactions that require precise conditions [2].

The behavior of cyclodextrin–surfactant systems in response to various stimuli—such as light exposure, pH changes, or specific chemical compounds—is actively being investigated to enable reversible and controlled modulation of the properties of these supramolecular aggregates. The addition of α-amylase to surfactant–CD systems leads to the degradation of the CD molecule, resulting in the release of surfactant molecules from the cyclodextrin cavities [2,144]. This subsequently triggers the self-assembly of the released surfactant molecules. Elevated levels of α-amylase are closely associated with acute pancreatitis; therefore, such α-amylase-triggered self-assembly systems may have potential applications in the early diagnosis and clinical treatment of this disease [144].

Research also includes the use of light-sensitive inclusion complexes of surfactants containing photoreactive groups [144,162], as well as modulation via changes in the electrochemical potential of solutions containing redox-responsive surfactants [163], and the introduction of macromolecules into the system with tunable interactions with the surfactant.

An interesting indirect application of cyclodextrin–surfactant inclusion complex formation was reported in a study where the authors developed a simple fluorescence-based method for the selective detection of anionic surfactants in water. The method relies on the disruption of a specific fluorescent complex formed between the hybrid chromophore styryl(pyridinium)-chromene and 2-hydroxypropyl-β-cyclodextrin. When anionic surfactants are added to the system, they cause a significant decrease in fluorescence intensity by displacing the chromophore from the cyclodextrin cavity and thereby disrupting the complex [164].

In addition to these practical applications, inclusion complexes formed by cyclodextrins and surfactants are of significant scientific interest. They provide a versatile model system to study supramolecular interactions, hydrophobic effects, and the specific roles of surfactant chain length and headgroup chemistry in complexation. The ability to modulate these complexes in response to stimuli such as light, pH, or enzymes offers insight into controlled self-assembly processes. These features make CD–surfactant systems highly relevant for targeted drug delivery, stabilization of labile compounds, development of stimuli-responsive materials, and the design of sensor systems for environmental or biomedical applications.

## 6. Conclusions

Cyclodextrins are capable of forming inclusion complexes with surfactants, where the stoichiometry depends primarily on the size of the cyclodextrin cavity and the structure of the surfactant, including the length of the hydrocarbon chain and the type of polar head group (ionic or nonionic, such as polyoxyethylene chains). When these inclusion complexes remain in the bulk aqueous phase rather than accumulating at interfaces, the formation equilibrium can be described by principles of detailed balance, which relate the rates of complex formation and dissociation. In such cases, tensiometric measurements can be used to determine the equilibrium constant, because the presence of inclusion complexes affects the surface tension of the solution.

At higher surfactant concentrations above the critical micellar concentration, and in the presence of sufficient cyclodextrin, the inclusion complexes can self-assemble into supramolecular aggregates. In these aggregates, hydrogen bonding may occur between the hydroxyl groups of cyclodextrins, either directly or mediated by water molecules, stabilizing the aggregate structure. This demonstrates a continuum from simple 1:1 inclusion complexes in solution to larger supramolecular assemblies, highlighting the versatile behavior of cyclodextrin–surfactant systems.

## Figures and Tables

**Table 1:** Overview of the most common cyclodextrin modification reactions and the resulting products.

Type of Reaction	Reaction Details	Resulting Products
Substitution	Monosubstitution at OH groups (positions 6, 2, or 3)	Mono-substituted CDs
	O-substitution	Methylated CDs, Hydroxypropylated CDs, Halogenated CDs
	Disubstitution	Di-substituted CDs (e.g., Di-thiolated α-CDs)
	Per-substitution	Per-substituted CDs (e.g., Per-halogenated CDs, Per-thiolated CDs)
	Random substitution	Randomly methylated β-CDs
Condensation	Condensation with propylene oxide	2-Hydroxypropylated CDs
	Polycondensation with epichlorohydrin or polyepoxides	Branched polymeric CD materials
	Formation of polymeric CD derivatives	PEG–βCD, Dextran–βCD, CD-based polyrotaxanes
Esterification	Acetylation	Peracetylated CDs
Oxidation	Introduction of aldehyde groups	Aldehyde-functionalized CDs

**Table 2:** Key aspects of cyclodextrin–surfactant complexation.

Cyclodextrin	Surfactant	Parameter/Condition	Result	Ref.
α-CD	Alkyl sulfates (C6–C12)	Binding constant (logK)	logK increases with alkyl chain length; α-CD forms stronger complexes than β-CD (e.g., C8: logK α-CD ≈ 2.6 vs. β-CD ≈ 1.9)	[113,116]
β-CD	SDS	Binding constant (logK)	logK ≈ 2.0 (calculated from CMC increase from 8.2 mM → 15 mM)	[140,144]
β-CD	Cationic surfactants (DTAB, CTAB)	Binding constant (logK)	β-CD–DTAB: logK ≈ 2.3; β-CD–CTAB: logK ≈ 3.1	[141,145]
Hydroxypropyl-β-CD	SDS + DTAB mixture	Temperature-dependent self-assembly	Worm-like micelles at 25 °C; vesicles at 50 °C	[151]
γ-CD	Alkyl ether carboxylates	Charge variation (pH)	Complexation and supramolecular aggregates form spontaneously; multilayer periodicity ≈ 10 nm	[149]
β-CD	C12E5, C12E10, C14E5, C16E5	Complexation behavior/Free CD fraction	β-CD forms complexes with all studied surfactants; fraction of free β-CD ranges from ~5% (C12E5) to ~95% (C16E5) depending on surfactant chain length and ethoxylation; vesicular structures are observed in some cases	[150]

## Data Availability

Not applicable.

## Abbreviations

The following abbreviations are used in this manuscript:

CMC	critical micellar concentration
CD	cyclodextrin
